# To Screen or Not to Screen? At Which BMI Cut Point? Comment on “Obesity and BMI Cut Points for Associated Comorbidities: Electronic Health Record Study”

**DOI:** 10.2196/37267

**Published:** 2022-06-29

**Authors:** Chrissa Sioka

**Affiliations:** 1 Department of Nuclear Medicine University Hospital of Ioannina Ioannina Greece

**Keywords:** obesity, body mass index, BMI, risk factors, screening, health services, chronic disease, heart disease, myocardial perfusion imaging, anxiety, depression

In their article, Liu et al [1] investigated if there are BMI cut points for various obesity-associated comorbidities. For this purpose, they evaluated 243,332 patients aged 18 to 75 years, documented in the electronic health record, who had at least 3 in-person clinical visits over a 2-year period. The authors reported a significant correlation and calculated cutoff points of BMI with 6 comorbidities, including coronary artery disease, hypertension, hyperlipidemia, obstructive sleep apnea, osteoarthritis, and type 2 diabetes mellitus [1]. Interestingly, no association was found with anxiety and depression.

In our recent study [2], although of a different concept, we prospectively evaluated a cohort of 80 patients who were subjected to myocardial perfusion imaging for myocardial ischemia evaluation. All patients in the study were additionally evaluated for the presence of anxiety and depression. Furthermore, cardiological risk factors, including obesity, were additionally assessed. Like the study by Liu et al [1], we found a positive association between obesity and myocardial ischemia. However, we also found a correlation between obesity and depression/anxiety and myocardial ischemia [2]. Other studies have also reported obesity and depression/anxiety as independent risk factors for acute coronary syndrome in young women [3] and that female patients had more central obesity and greater anxiety than male patients with coronary artery disease [4].

In any event, I agree with the conclusions of Liu et al [1] that additional studies may be needed to establish which comorbidities need to be screened in patients with overweight for appropriate management.
